# Community Cancer Champions’ Project: Learning From the Design and Implementation of an Integrated Health and Voluntary and Community Sector (VCS) Asset-Based Community Development Project – A Case Study From Plymouth, England

**DOI:** 10.5334/ijic.9054

**Published:** 2025-10-10

**Authors:** Katy Stevenson, Felix Gradinger, Niqui Bond, Debbie Freeman, Richard Byng

**Affiliations:** 1 Community and Primary Care Research Centre (CPCRC), Faculty of Health, University of Plymouth, 6 Research Way, Plymouth Science Park, Derriford, Plymouth, Devon, PL6 8BU, UK https://www.plymouth.ac.uk/research/primarycare; 2 National Institute for Health Research (NIHR) Plymouth Health Determinants Research Collaboration (PHDRC), Public Health, Plymouth City Council, Tailyour Road, Plymouth, PL6 5DH, UK; 3 Community and Primary Care Research Centre (CPCRC), Faculty of Health, University of Plymouth, 6 Research Way, Plymouth Science Park, Derriford, Plymouth, Devon, PL6 8BU, UK https://www.plymouth.gov.uk/plymouth-healthdeterminantsresearch-collaboration-phdrc; https://www.plymouth.ac.uk/research/primarycare; 4 Zebra Collective, Mount Wise Neighbourhood Centre, 75 Clowance Street, Plymouth, PL1 4NH, UK https://zebra.coop/; 5 Community and Primary Care Research Centre (CPCRC), Faculty of Health, University of Plymouth, 6 Research Way, Plymouth Science Park, Derriford, Plymouth, Devon, PL6 8BU, UK; 6 National Institute for Health Research (NIHR) Applied Research Collaboration South West Peninsula (PenARC), UK https://www.plymouth.ac.uk/research/primarycare; https://arc-swp.nihr.ac.uk/

**Keywords:** cancer, health inequity, embedded research, qualitative, ethnography

## Abstract

**Background:**

Cancer survival rates vary significantly between low and high-income areas. By leveraging community assets, healthcare inequities may be addressed. Nationally, Macmillan Cancer Support (Macmillan) (a national cancer charity) is working with local Voluntary and Community Sector (VCS) organisations to improve cancer care.

In Plymouth, where cancer mortality is above average, Macmillan has partnered with the Zebra Collective (Zebra) (a community cooperative), Age UK Plymouth (a local charity), The Wolseley Trust (Social Prescribing team), and General Practice (GP) surgeries. In Spring 2024, the Plymouth Cancer Champions’ Project (PCCP) launched to address these inequities through community-led approaches via peer-to-peer community engagement and volunteer recruitment.

**Approach:**

This Integrated Care Case is a practice-based account of how through an embedded ethnographic action research approach, a small community cooperative (Zebra) is influencing its’ local low-income community’s understanding of and engagement with cancer care services from an asset-based community development approach.

**Findings:**

The PCCP prioritises involving individuals with lived experience, including those from lower socio-economic status backgrounds, minoritised ethnic groups, and cancer-affected backgrounds, in leadership roles. This collaborative, community-driven approach fosters inclusivity, empowerment, and engagement, and a deep contextual understanding of the community context including barriers and strengths. Through an innovative asset-based community development approach, the deficit narrative is countered- enabling people-led change, influence and learning within cancer care inequity and integrated care.

## Introduction

The global cancer burden is projected to reach 28.4 million cases by 2040, representing a 47% increase compared to 2020 [1]. The rapidly increasing burden is driven by population aging, shifts in exposure to risk factors, many of which are linked to socioeconomic development [2]. Global estimates reveal striking inequities in the cancer burden both between high- and low-income countries, as well as between high- and low-income areas within countries [3,4]. Cancer care should be accessible and equitable for all, regardless of economic status. However, the reality reveals persistent barriers to achieving this, as socioeconomic disparities in cancer survival rates remain prevalent [5].

The drivers of worse cancer outcomes in low-income areas are multifaceted and include higher prevalence of lifestyle risk factors such as smoking, obesity, and alcohol consumption, which increase the risk of developing cancer [6]. Additionally, lower rates of participation in cancer screening programmes, delayed presentation with symptoms, and reduced access to diagnosis and treatment contribute to these disparities [7]. These factors collectively contribute to poorer cancer outcomes for lower-income communities, highlighting the need for targeted interventions to address both prevention and healthcare access within these populations. Rising levels of multimorbidity call for integrated commissioning strategies that incorporate collaboration with the VCS sector [8].

Despite free at the point of delivery healthcare, accessible screening programmes, and recognisable referral pathways, deaths from cancer equate to just over a quarter of all deaths in England in a typical year, and mortality rates from cancer remain the highest in the most deprived areas of the country [9,10].

UK-based studies examining cancer disparities in low-income populations from 2010 onwards highlight several key factors that exacerbate cancer inequities within these communities. For individuals from minoritised ethnic groups, themes such as structural racism within the NHS, unmet cultural, religious, and language needs, and barriers faced by newcomers to the UK contribute to decreased participation in cancer screening programmes. These factors, along with a lower likelihood of presenting with cancer symptoms, significantly hinder access to timely diagnosis and treatment, further compounding health disparities [11,12,13].

In low-income communities, there is also often a lack of awareness or understanding regarding the importance of cancer screening, or a false perception of being at low risk for cancer [14]. Knowledge of cancer risks, signs and symptoms is lowest and actual presentation time is longest in lower socio-economic groups [15].

Individuals from lower socio-economic groups are also more likely to experience immediate stress and fear-based responses when experiencing new signs and symptoms of potential cancer, and when compounded with other survival priorities, this fatalistic processing contributes to later presentation which in turn creates worse outcomes [16].

The evidence also highlights experiences of mistrust or embarrassment related to healthcare, often stemming from negative past encounters [17]. Further systemic barriers- such as transport difficulties, limited services, lack of support, and referral delays can hinder access to cancer screening and care [18,19,20,21,22].

Despite multiple public health and UK-government funded initiatives to mitigate against cancer inequities, unfortunately, they prevail [23,24]. There are increasing schools of thought that instead of taking a top-down approach that by co-creating, and co-developing approaches by capitalising on the capabilities and assets of those living within lower-income communities, healthcare inequities might be reduced [25,26,27].

Integrated care, often referred to as integrated health, coordinated care, or comprehensive care represents a global movement in healthcare reforms crucial for cancer care [28]. It emphasises new organisational models designed to provide more cohesive and interconnected wider public services [29]. The VCS sector plays a unique and vital role in integrated care, as well as community engagement, person-centred care, and addressing health inequities. By being deeply embedded within local communities, VCS organisations build trust and foster connections with individuals who may be marginalised or underserved by traditional healthcare systems and have the potential to capitalise on peer support dynamics [30]. Their presence allows tailored, culturally sensitive support, ensuring person-centred care, while also bridging communities with formal health services and advocating for vulnerable groups [31]. Through targeted initiatives, the VCS can help to reduce barriers to access, address social determinants of health, and work towards greater equity in health outcomes, making them an indispensable partner in achieving inclusive, integrated, holistic care [32].

In recent years, Macmillan launched its Community Cancer Champions Programme [33]. This Integrated Care Case (ICC) complements other evaluations and presents emerging findings from a pilot programme in one locality exploring how a community cooperative, in partnership with VCS organisations and primary care, addresses cancer outcomes in low-income communities. Using asset-based community development and embedded ethnographic action research, it examines how these collaborations influence engagement with cancer prevention, screening, diagnosis, and support, aiming to identify strategies to improve health equity and access to care.

## Description of the Plymouth Cancer Champions Project

### Setting

Plymouth, a coastal city in the South-West of England was one of Macmillan’s fourteen chosen pilot sites for this programme [34]. Plymouth ranks among the 20% most deprived cities in England, with Devonport and Stonehouse (two areas in Plymouth) among the 1% most deprived areas nationally [35]. In keeping with other areas of deprivation, the mortality rate for all cancers combined in Plymouth is significantly higher than the England average [36]. In Summer 2023 the Plymouth Cancer Champions Project (PCCP) launched its’ three-year project, funded by Macmillan and hosted by Zebra in close collaboration with community VCS and primary care partners.

### Design of the Plymouth Cancer Champions Project

Zebra is a small, community cooperative and collective based in Devonport, who are embedded within the local community and are committed to their work in culture change and asset-based community development (ABCD) [37]. ABCD approaches emphasise the strengths and capacities already present within a community, rather than focusing on deficiencies or needs [38]. This method recognises and builds on local resources, such as individual skills, associations, physical assets, and shared experiences, to foster sustainable community development. ABCD encourages the involvement of residents in identifying and mobilising these assets, which helps to empower communities and improve well-being by shifting the focus from problems to solutions [39]. The approach holds several key principles such as building relationships, identifying local leaders, and connecting community assets to tackle challenges collectively [40]. The PCCP works informally with multiple VCS groups and more formally in collaboration with:

– Age UK Plymouth: a local charity which exists to care for and work with older people and their carers’ in and around Plymouth [41].
– The Wolseley Trust: a community trust which helps to bring improvements in health and finances to people living in inner-city Plymouth via social prescribing [42].
– Waterside Health Primary Care Network (PCN): a group of General Practice (GP) surgeries covering the Devonport and Stonehouse areas in Plymouth [43].
– A University of Plymouth embedded GP trainee and researcher.

### Evolution of the PCCP

In early 2023, Macmillan began discussions with Zebra, followed by consultations with Plymouth-based organisations. Agreed themes led to a multi-partner, single-project approach. The PCCP board was formed in August 2023, including representatives from Macmillan, Zebra, The Wolseley Trust, Age UK Plymouth and the Waterside Health PCN. Their shared goal: to address cancer inequities in Plymouth, starting in Devonport and Stonehouse.

Over Autumn 2023 they formulated roles, responsibilities, a memorandum of understanding, and embedding an ABCD approach within the PCCP. By January 2024 the PCCP Project Manager was recruited and by March 2024 the various PCCP team members were recruited including a Social Prescribing Link Worker (The Wolseley Trust), Community Engagement Lead for Older People (Age UK Plymouth), Community Engagement Lead for Place-Based (Zebra), Cancer Champions Coordinator (Zebra) and Digital Inclusion Support Worker (Zebra).

All team members underwent training delivered by Zebra, Macmillan, Age UK Plymouth and the embedded researcher, to foster a culture of strengths-based collaboration. By May 2024 the team were out in Devonport and Stonehouse making links with people and community groups. From June 2024, the PCCP opened its’ doors to the community hosting several Cancer Awareness, Kindness and Empathy (CAKE) and Cuppa cafés around the city, as well as attending other community groups and organisations to spread the word about the PCCP [44]. From November 2024 the PCCP team started to recruit PCCP volunteers; *“Cancer Champions”-* people who are happy to have everyday conversations about cancer and offer a bit of support to members of their community, often with their own personal experiences of cancer. Please see **Appendix 1: Infographic of the Evolution of the Plymouth Cancer Champions Project** for a more detailed overview of the project.

While all team members contribute to community engagement, signposting, event planning and delivery, and evaluation, their specific roles are outlined below:

- Project Manager (Zebra): Coordinates the team, leads evaluation, and liaises with local organisations and community stakeholders.
- Cancer Champions Coordinator (Zebra): Recruits, trains, and supports volunteer *“Cancer Champions”*.
- Community Engagement Lead (Place-Based) (Zebra): Drives integration and outreach, focusing on minoritised ethnic groups.
- Digital Inclusion Support Worker (Zebra): Helps individuals overcome digital barriers to access health services.
- Community Engagement Lead (Older People) (Age UK Plymouth): Supports older adults facing cancer and health inequities, improving group access and participation.

Social Prescribing Link Worker (The Wolseley Trust): Provides personalised support for cancer patients in partnership with local GP surgeries.

## Methodology

This research study complements an ongoing standard reporting led by Zebra on behalf of Macmillan, as well as a national evaluation looking at the Macmillan Cancer Champions’ Programme.

### Research question

Following a multi-organisational, integrated asset-based community development approach, how can a community collective influence engagement of community members, living in low-income communities, with cancer education and services?

### Aim

To explore how the Plymouth Cancer Champions’ programme (PCCP) influences low-income Plymouth communities’ engagement with cancer prevention, screening, diagnosis, treatment, and support services.

### Role of the researchers

The Principal Investigator (PI) (KS) is a trainee academic GP with experience of primary care, community research, and has lived and professional experience of Plymouth’s low-income communities, they are working with colleagues from the National Institute for Health and Social Care Research Health Determinants Research Collaboration Plymouth (NIHR HDRC) including Researcher in Residence (FG) and the Community and Primary Care Research Group and NIHR PenARC Director (RB), in collaboration with Zebra team members including the Project Manager of the PCCP (NB), and Community Projects Lead (DF). By embedding the PI into the PCCP, the focus aims to shift research into applied settings of contextual relevance, solving practical problems by fostering collaboration, knowledge sharing, linking theory and action through cycles of reflection, and reducing power differences in turn combining relevance and rigour [45]. Embedded research can assist in the development of more effective services, by encouraging greater interaction between those planning, delivering and evaluating services and the researchers [46]. Due to the nature of the PCCP, and its role in this collaborative community initiative engaging VCS and primary care, it lent itself to taking on an embedded researcher to observe, co-design, co-deliver and co-evaluate the work in a community-engaged manner, with the aim to enhance the development of an evidence-base and initiative. The PI holds an honorary contract with Zebra.

### Ethnographic action research

By taking an ethnographic action research approach the PI has been able to study the interactions of the participants within everyday opportunistic, naturally occurring events in addition to research-specific contexts for data collection [47]. The PI has observed the formation of the PCCP board, the recruitment of the PCCP team and *“Cancer Champions”*. The PI has collaborated with the PCCP board and team to co-design, co-deliver education, training and group reflective sessions to PCCP team, and co-evaluate the PCCP. The PI has continually fed back to develop the PCCP from their perspective as a clinician and embedded researcher. The PI has been able to simultaneously influence, and induce change, through the synergistic process of getting involved whilst undertaking research and evaluation. This research study is embedded within the ongoing PCCP, so participants are aware of the ongoing recruitment process as the PI has forged working relationships with them.

### Participant recruitment

Purposive recruitment was undertaken to gain greater insights from a variety of perspectives to identify common themes across the sample [48]. Participants included people involved as PCCP board and team members. Participants were contacted either via email, or in person, were given a Participant Information Sheet and a written Participant Consent Form. All ten members of the PCCP board including representatives from Zebra, Macmillan, Age UK Plymouth, The Wolseley Trust, and the Waterside Health PCN were recruited. Seven PCCP team members including the Project Manager, Cancer Champions Coordinator, Community Engagement Lead (Place-Based), Digital Inclusion Support Worker, Community Engagement Lead for Older People (Age UK Plymouth) and Social Prescribing Link Worker (The Wolseley Trust) were recruited. One member of the PCCP board, and another member of the PCCP team left their roles during the project, but their new counterparts were subsequently recruited into this research study.

To establish trust, the PI spent over 80 hours during 18 months with Zebra to allow participants to become familiar with their presence. This continuity helped reduce perceptions of the researcher as an outsider and trust was nurtured by being transparent about the research objectives, processes, and how the data would be used. Participants were encouraged to ask questions, express concerns, and contribute to the direction of the study. The PI practised reflexivity to recognise and minimise their own biases and power dynamics. As action research emphasises co-creation, participants were treated as collaborators rather than subjects. Involving them in decision-making and iterative reflection built mutual respect and a shared sense of purpose.

### Data collection procedure

Data were gathered in an unstructured manner from a variety of sources, including non-structured observations and naturally occurring events such as meetings, job descriptions, participant reflections, educational training sessions, and opportunistic interviews [49]. The PI engaged directly in board meetings, training sessions, reflective group discussions, and community events to gain firsthand insight into participants’ routines, interactions, and environments. Observational data was documented through detailed field notes and a reflexive journal, which captured key observations, direct quotes, and the PI’s evolving interpretations. Relevant documentation was also curated throughout the process to support and contextualise the observations.

To facilitate more in-depth study, two semi-structured focus groups with the PI were hosted- one focus group of team members (six participants) and one focus group of board members (five participants) were facilitated. A guide was used to facilitate discussion and was provided to participants in advance of the focus group (please see **Appendix 2: Focus Group Interview Guide**). The focus groups were audio-recorded, and the PI simultaneously took notes.

### Data analysis

Data were collected from naturally occurring events, no findings were predicted, in keeping with an inductive approach to gain a deep understanding of the perspectives of the participants [50]. The focus group transcripts were read by the PI in conjunction with the Researcher in Residence (FG); initial codes were generated, then re-reviewed for themes and sub-themes with other members of the research team, these were then shared with participants drawing upon the method of constant comparison and member checking [51].

### Lived experience

The PCCP emphasises the inclusion of individuals with lived experience, with board members and team members representing diverse backgrounds, including lower socio-economic status groups, the Gypsy, Roma, and Traveller community, from minoritised ethnic groups, and those with personal experiences of cancer or chronic illness.

### Ethics

Ethical approval was granted by The University of Plymouth’s Ethics Committee in March 2024 (PEOS #4866).

## Results and discussion

The following results are derived both from the ethnographic data, as well as focus group data with both the project board and team. Ethnographic data was collated from:

- Project board meetings from March 2024 to May 2025
- Meetings with various board and team members
- Observation, participation in, and delivery of team training sessions and reflective group sessions
- Participation in and observation of CAKE and Cuppa cafés
- Review of relevant documentation, including job descriptions, participant reflections and Zebra and Macmillan evaluation data

### Zebra local reporting data (for Macmillan)

Since June 2024, the PCCP team have hosted 25 CAKE and cuppa sessions with a total of 203 people in attendance, they have facilitated 11 *“Cancer Champion”* awareness sessions and have trained 71 *“Cancer Champions”*, the Social Prescribing Link Worker has supported 56 individuals with cancer, the Community Engagement Lead for Older People (Age UK Plymouth) has supported 47 different individuals, and the Digital Inclusion Support Worker has delivered three digital group workshops run with 29 people in attendance and the PCCP team have made 1057 signposts to different sources of support for individuals. The team also hosted a Cancer Champions Community Connections event with 95 delegates from a variety of professional backgrounds and from the local community.

### Foundations, team dynamics and collaboration

This theme sets out the project’s architecture, values, and collaborative framework. The establishment of the PCCP was rooted in strong collaborative partnerships and a shared vision for reducing cancer care inequities. The initial alignment between Macmillan and Zebra emphasised embedding ABCD principles, which required buy-in from all board members. Reflexivity on values, health inequity, and lived experiences added depth and cohesion, shaping the board’s approach.

This collaborative ethos extended to the recruitment of the PCCP team, reflecting the diversity and lived experiences of the communities served. An induction programme, led by key partners, grounded the team in cancer care, health inequity, ABCD approach, and trauma-informed practices, fostering an inclusive, solutions-focused culture.

The inclusion of individuals with lived experience on the PCCP board, in the team and emerging volunteer workforce is a hallmark of this initiative, bridging the gap between theory and practice.

*“I think the fact that you’re from India and you are of ethnic minority means you’ve really managed to get into some of those groups. Perhaps… they embrace you in a way that I don’t think they would embrace me.”- ((NB) Focus group participant)*

Observing how lived experience shaped the PCCP highlighted a profound shift in power dynamics within healthcare innovation, traditional hierarchies gave way to mutual respect and shared learning [52]. For a global audience, this serves as a reminder that inclusivity and respect for lived experience are not mere add-ons but core drivers of meaningful innovation. Limitations expressed by this approach did include initial difficulties navigating internal organisational complexities and multi-organisational working patterns, as well as support for all organisations in taking on an ABCD approach.

### Community context: understanding barriers, needs and strengths

This theme showcases the community context – the social realities and barriers the project seeks to navigate. Although Zebra is well known in Devonport and Stonehouse, the project team dedicated several months to understanding the dynamics of the communities by engaging with community groups centred around identity, such as religious organisations and services for asylum seekers and refugees, as well as activity-based groups like knitting circles and walking football teams.

Like the efforts of Lansing et al. in the USA, the PCCP prioritised fostering trust between its team and local community members, working at the pace of trust is crucial for establishing healthy, mutual relationships, creating safe spaces, promoting transparent communication, and advancing equity [53].

Through the Cancer Awareness, Kindness and Empathy (CAKE) and Cuppa cafés, discussions in other community groups and at community events, as well as targeted conversations with cancer patients with the Wolseley Trust Social Prescribing Link Worker and the Age UK Community Engagement Lead the project team prioritised listening to the community’s needs rather than presuming areas for intervention, thus key priorities emerged.

Key areas included enhanced support for over-65s, housebound individuals, carers, the bereaved, minoritised ethnic groups, those facing financial or housing instability, mental illness, or substance misuse. People highlighted the need to feel heard by healthcare professionals, build trust, improve cancer awareness (especially around women’s health), access primary care more easily, improve digital inclusion and transport, and receive better post-diagnosis support.

*“…one of the things I wasn’t quite expecting to be so prominent is how people’s own health always seems to take them back step because they’re trying to put everything else first and sometimes, they just don’t have the mental capacity to think about their own health.”- (‘J’ Focus group participant)*

The PCCP’s adoption of ABCD and its partnership with diverse stakeholders highlights a shift from top-down interventions to participatory, bottom-up approaches. This approach is innovative in its emphasis on leveraging local strengths and fostering co-creation with a strong focus on embedding within the local community, reaching out to other community groups and organisations.

Some of the challenges included variable attendance at community events as well as managing group dynamics. The team were also required to handle emotionally challenging personal narratives, ensure appropriate signposting to support services, and engage with individuals experiencing health inequities not exclusively related to cancer.

### Asset-Based Community Development (ABCD) Approach

The ABCD model underpins how the project works in and with communities, countering deficit narratives and enabling people-led change. The team continually tried to take on an ABCD approach, reflecting not only on barriers but on individuals’ strengths, likes, passions and hopes to support self-care, management, reintegration, upskilling and navigation of their own lives and journeys.

*“So sometimes the in terms of the asset-based stuff is when you’re looking to kind of find strength. Both the strengths that exist within communities, whatever they may be, but also more importantly in people. We’re often not discovering strengths…I’m sometimes just a mirror that helps someone …to see that they have got a bit of power in their lives, whether they feel they have or not.” – (‘K’ Focus group participant)*

One such story involved a team member speaking with an elderly housebound woman who had been diagnosed with a terminal illness and isolated herself due to fear and anxiety. After suggesting that she attend a nearby community group, the woman agreed and was warmly welcomed by group members who remembered her from years ago. This experience prompted a reflection on the meaning of being “housebound,” emphasising how empowering invitations like “I’m going, do you want to come?” can encourage individuals to overcome isolation. By offering a non-pressured invitation with an ‘out’ clause, the team member helped the woman reconnect with her community, demonstrating the importance of empathy, connection, and challenging labels.

Additionally, the PCCP team extended their outreach to minoritised ethnic communities, including engagement at a local mosque. Following a session, one woman felt more able to share her breast cancer journey with her peers, which led to others’ self-examining, and further engagement with the local breast cancer screening team who have delivered a subsequent outreach session to the Islamic community. This aligns with global approaches in which faith-based organisations have encouraged cancer screening through outreach programmes, utilising the trust and influence they have within their communities [54].

**Figure 1:**
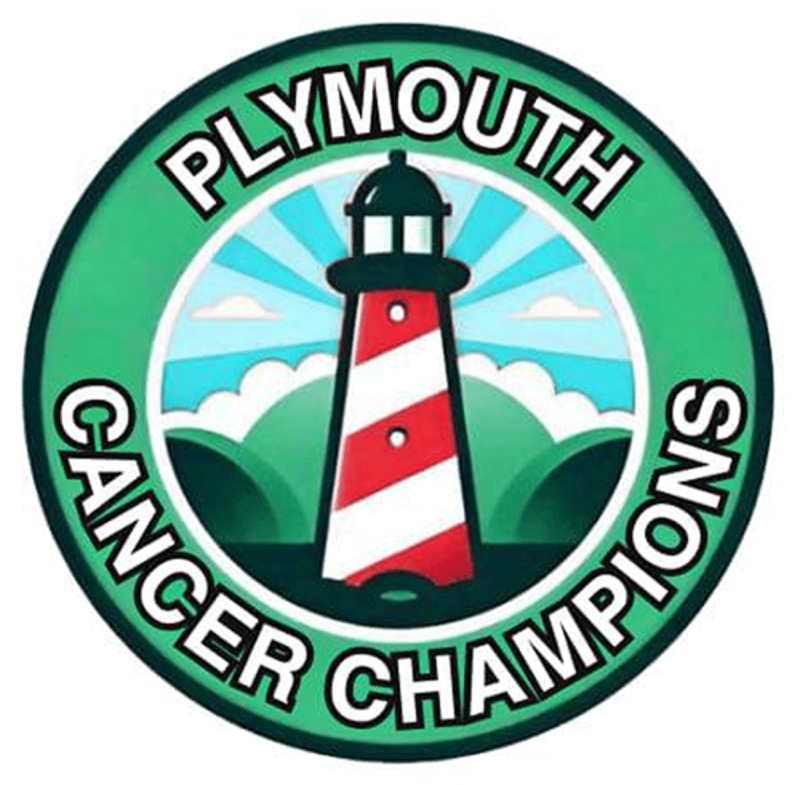
Plymouth Cancer Champions’ Logo.

Another notable example involved a community member skilled in digital design, who contributed to the project by creating the PCCP logo (see Figure 1 below). This act of creativity not only enhanced the project’s visual identity but also empowered the individual by acknowledging their talent and allowing them to contribute meaningfully to the initiative.

Similarly, a tailor from a migrant background, with minimal English and with a diagnosis of cancer, had previously faced isolation and struggled with mental health challenges, found a sense of belonging and purpose through his involvement in a sewing circle after signposting from the PCCP team. The tailor’s background in sewing allowed him to take a leadership role in the group, significantly improving his mental health and reducing his feelings of isolation.

These examples highlight the core principle of ABCD: when individuals’ skills and assets are recognised and utilised, it fosters a sense of empowerment and can lead to profound personal transformation.

As part of the ABCD approach, the team recruited *“Cancer Champions”-* trusted community volunteers who help reduce fears, dispel myths, and share key cancer messages. Their roles include peer support, accompanying others to GP appointments, promoting screening, running events, and aiding digital access. Many have found the role boosts their confidence and enables them to advocate for others.

By tapping into the resources and knowledge within the community and focusing on empowerment, social inclusion, and accessibility, the PCCP may have fostered an environment where meaningful change could take place. This approach demonstrated that, when communities are supported to identify and utilise their own assets, the potential for innovation is not only limitless but also deeply rooted in the lived experiences and capabilities of the individuals within those communities.

A key limitation has been maintaining a genuine ABCD approach across organisations, particularly in aligning working practices and ensuring *“Cancer Champions”* reflect the communities they serve.

*“I just wanted to kind of bring up to the asset-based community development work is… I think it’s deeply, deeply counter cultural, both in on a societal level and in the world that we work in.” (‘D’ Focus group participant)*

### Influence and learning

This theme outlines the project’s supposed influence and how it understands itself, it captures both the tangible and intangible results of the work, including perceived shifts in individuals, services, and systems. Taking an ABCD approach, the PCCP project has suggested that community collectives can play a pivotal role in fostering meaningful engagement with cancer education and services among residents of low-income communities.

Central to this potential for culture change is the cultivation of community connections- a recurring theme manifested in informal gatherings such as CAKE and Cuppa cafés events, peer-led groups, and grassroots initiatives. These gatherings serve as accessible, familiar entry points that may lower barriers to engagement by embedding health-focused conversations within existing social networks.

Community members who attended these events multiple times often developed deeper relationships and a sense of belonging, which in turn nurtured openness to learning and discussing cancer-related issues. The presence of professionals at community events further may have enhanced trust and demystified the health and social care systems. This helped foster a shared understanding of cancer and its impacts, particularly in minority communities where cultural or linguistic barriers might otherwise impede access to information.

The influence of the project was also evident in how it supported peer support structures- from making a drink to offering a listening ear- which proved vital in reducing isolation, especially for those navigating a new or ongoing cancer diagnosis. In practical terms, Zebra acted as a bridge between individuals and formal services, supporting the navigation of healthcare systems, facilitating access to screening, and offering social prescribing to relevant groups. The project also addressed structural barriers, such as the cost of travel to hospital appointments, and made health services more approachable by enabling community members to interact with *“Cancer Champions”* from similar backgrounds. Zebra helped residents reclaim agency over their health through increased knowledge, changing identities, and more informed choices, reinforcing the idea that health is a communal, not solely an individual endeavour.

Furthermore, the project’s influence extended beyond the individual to the systemic level by engaging with the Waterside PCN, supporting activities like mass messaging to remind patients about screening, translating screening materials into different languages, reinforcing cancer awareness campaigns coding patients discharged from secondary cancer services to prioritise support from those no longer formally engaged with health services. This multi-level, relational approach to community engagement underscores the transformative potential of ABCD: by valuing and mobilising existing community assets, the project may not have only enhanced access to cancer education and services, but also contributed to community empowerment, sustainability of health messaging, and ultimately, life-saving outcomes.

*“He was telling someone about his cancer experience. And as a result, they then went and had a test and they found out they had cancer …and he sort of went…my gosh, this talking about cancer really saves lives, doesn’t it?”- (‘N’ Focus group participant)*

The integration of social prescribing and digital inclusion initiatives within the PCCP demonstrated how small, community-centred changes can improve accessibility and engagement. Social prescribing, which connects people to local community resources and activities, helped address a wide range of social and health issues [55]. By encouraging people to participate in activities that promote social interaction, physical activity, and emotional support, the PCCP showed potential to address isolation and improve overall well-being. The focus on digital inclusion was particularly impactful in increasing accessibility for community members who had previously been excluded from online resources. By empowering individuals with these skills, the PCCP was able to bridge gaps in digital access, ensuring that more people could benefit from online healthcare services and information [56].

### Contextualising and addressing the challenges of integrated care

Integrated care, as hinted at in the PCCP between healthcare, and VCS organisations, represents a holistic approach that bridges gaps between healthcare services, community resources, and lived experiences. This work from Plymouth aligns with efforts to address health inequities by blending community-led initiatives with formal healthcare systems nationally [57]. Observing the PCCP underscores the potential ABCD in fostering integrated care; a community-led framework- when paired with strategic partnerships like those between Macmillan and local VCS organisations have the potential to address structural barriers and enhance healthcare equity, emphasising the necessity of tailoring healthcare delivery to the socio-economic and cultural contexts of underserved populations.

### Implications for policy

Integrated care often exposes systemic barriers like organisational silos and limited resources and the research revealed some specific practices which could be promoted through policy. The PCCP addressed such barriers through collaborative, multi-partner leadership while also creating a unified single project approach. Building trust among stakeholders- community groups, healthcare providers, and residents- was essential but the challenging nature of the task indicates a need for resourcing this ongoing function. We also propose that policy should emphasise that successful integration requires a cultural shift toward collaboration and shared responsibility for health outcomes. Achieving this may require capacity-building, especially in low-resource settings.

### Implications for practice

As a practice embedding and supporting community champions is a relatively low-cost, adaptable innovation. Meaningful community engagement is possible without heavy resource demands, strengthening training, digital inclusion, and community engagement alongside community champions has the potential to help address not just cancer but a range of health inequities.

### Implications for research

Embedded ethnographic action research provided ongoing, context-rich insights into community needs. This approach captured the community’s daily realities, social dynamics, and barriers to cancer care. it has the potential for both generating immediate local impact (due to contextual relevance) and also more widely applicable insights. The method allowed for continuous adaptation based on community feedback, it ensured local changes to health systems were evidence-informed, culturally sensitive, making them more likely to be impactful.

## Scope for improvement

While the PCCP has made promising strides in community engagement and innovation, there are several limitations to consider. First, the scope of the project was limited to specific geographic areas, which may affect the transferability of the findings to other communities. Additionally, despite efforts to engage a diverse range of individuals, some minority groups or those with more complex health and social needs may have been underrepresented or less engaged due to various barriers such as language, mistrust of services or mental health issues.

Ongoing efforts are also focused on sustaining community involvement beyond the PCCP period, to ensure the long-term impact and continuity of the *“Cancer Champions”* initiative.

These limitations highlight the need for ongoing reflection and adaptation to ensure that future initiatives continue to meet the needs of diverse communities.

The next steps for the PCCP are expanding its geographic reach, increasing engagement with underrepresented groups, and continuing to collate and evaluate data. Scaling grassroots innovations and integrating the project with mainstream health services will help ensure sustainability.

## Lessons learned

- Value of living each other’s experiences- involving individuals with lived experience in both the project board, team and as “*Cancer Champions*” has been essential for creating meaningful connections and improving service delivery.
- Cross-sector collaboration- effective collaboration across diverse organisations and stakeholders, including community groups, healthcare providers, and support services, is critical for addressing complex health and social issues in an integrated manner.
- Community-centred approach- a key lesson learned is the importance of focusing on community strengths and assets rather than simply identifying needs, which fosters empowerment and engagement.
- Flexibility and reflexivity- the importance of being flexible and reflexive in practice- adapting based on community feedback and ongoing reflection-has been vital for maintaining relevance and ensuring the project meets the needs of the people it aims to serve.

## Conclusions

In conclusion, the PCCP has provided learning on the power of community-driven, asset-based approaches in addressing health inequities, particularly in cancer care. By leveraging grassroots innovation, personalised support, and meaningful community engagement, the project has hinted at positive and measurable change in the future. However, the integration of care in culturally and socio-economically diverse communities presents ongoing practical challenges- ranging from aligning services across fragmented systems to ensuring cultural competence and building trust with underserved populations. Addressing these complexities requires adaptive, context-sensitive strategies that are co-designed with communities, as well as ongoing, embedded learning. Moving forward, expanding the model’s reach, deepening cross-sector partnerships, and embedding mechanisms for cultural responsiveness and sustainability will be key to ensuring equitable, long-term improvements in cancer outcomes for low-income and marginalised groups.

## Appendix 2: Focus group interview guide

The Plymouth Cancer Champions’ Project: An embedded ethnographic approach to community-engaged action research

### Outline for focus group

- Introduce the focus group as an informal and relaxed discussion
- Ensure everyone has read the participant information leaflet and has completed the consent process (this was a long time ago)
- One-hour discussion about yourselves, your motivations to join the board, cancer care and inequity in Plymouth, Asset Based Community Development, project design and delivery, evaluation, embedded research and other reflections
- This will be recorded for data analysis purposes (via Microsoft teams and via iPhone)
- Anything you say will be anonymised in any future outputs and any identifiable information about yourself will be kept confidential
- Anyone can leave at any time without explaining why, but anything you’ve said up until then can’t be deleted but can be excluded from analysis on request
- In combination with the analysis of the observations and reflections, the analysis of the focus group will be transcribed, written up, analysed and used to inform research outputs which will be shared with yourselves
- The purpose of the focus group is to gather some more qualitative data around the project and to contextualise findings ie. the hows, whats, wheres and whys
- Feel free to build on each other’s experiences
- Please don’t talk over one another
- Respect one another’s’ opinions
- It’s more of a discussion than an interview
- I’ve put together a loose structure to follow but we can just flow as the conversation flows
- At the end we’ll be running through some debriefing forms

### Introductions

- Introduce yourself
- Your role
- Ties to the Plymouth Cancer Champions’ Project
- Reasons for participating in the research project

### Cancer care in Plymouth

What were your thoughts on cancer care including screening, prevention, diagnosis and support for people living in lower income areas in Plymouth prior to the project?

### Asset based community development work

What were your thoughts on asset-based community development prior to the project?

### Design and delivery

Does anyone want to share any areas of good practice during their experience of the Plymouth Cancer Champions’ Project? What are you most proud of? What stories would you like to share?

Does anyone want to share any areas that could have been made even better if you/we did what during your experience of the Plymouth Cancer Champions’ Project?

– What helped and what hindered you in achieving what you wanted to get out of it?
– You might want to think about the formation of your role, the board, the vision, memorandum of understanding, recruitment, managing the team, forward planning etc.

### Evaluation

#### Influence of the Plymouth Cancer Champions’ Project

What changes/difference did it make to you and the people you were in contact with? Do you think the Plymouth Cancer Champions’ Project has had on the selected communities in Plymouth?

Can you give specific examples of how the Plymouth Cancer Champions’ Project, and asset-based community development approach, has influenced community members?

Does anyone have any experiences involving stakeholders, employees, volunteers and community members that they would like to share with the group?

Why do you think it has had the above influence?

### Reflection

Reflecting on the project, what (if any) skills or learning have you developed? If so, how and why?

### Embedded research

What was your experience of working alongside an embedded researcher? What influence did they have on the project?
